# Reverse-engineering the Venus figurines: An eco-life-course hypothesis for the aetiology of obesity in the Palaeolithic

**DOI:** 10.1093/emph/eoae031

**Published:** 2024-11-28

**Authors:** Jonathan C K Wells, Frank L’Engle Williams, Gernot Desoye

**Affiliations:** Population, Policy and Practice Research and Teaching Department, UCL Great Ormond Street Institute of Child Health, London, UK; Department of Anthropology, Georgia State University, Atlanta, GA, USA; Department of Obstetrics and Gynaecology, Medical University of Graz, Graz, Austria

**Keywords:** obesity, life-course, diet, protein leverage, Venus figurines, Palaeolithic

## Abstract

Evolutionary perspectives on obesity have been dominated by genetic frameworks, but plastic responses are also central to its aetiology. While often considered a relatively modern phenomenon, obesity was recorded during the Palaeolithic through small statuettes of the female form (Venus figurines). Even if the phenotype was rare, these statuettes indicate that some women achieved large body sizes during the last glacial maximum, a period of nutritional stress. To explore this paradox, we develop an eco-life-course conceptual framework that integrates the effects of dietary transitions with intergenerational biological mechanisms. We assume that Palaeolithic populations exposed to glaciations had high lean mass and high dietary protein requirements. We draw on the protein leverage hypothesis, which posits that low-protein diets drive overconsumption of energy to satisfy protein needs. We review evidence for an increasing contribution of plant foods to diets as the last glacial maximum occurred, assumed to reduce dietary protein content. We consider physiological mechanisms through which maternal overweight impacts the obesity susceptibility of the offspring during pregnancy. Integrating this evidence, we suggest that the last glacial maximum decreased dietary protein content and drove protein leverage, increasing body weight in a process that amplified across generations. Through the interaction of these mechanisms with environmental change, obesity could have developed among women with susceptible genotypes, reflecting broader trade-offs between linear growth and adiposity and shifts in the population distribution of weight. Our approach may stimulate bioarchaeologists and paleoanthropologists to examine paleo-obesity in greater detail and to draw upon the tenets of human biology to interpret evidence.

## INTRODUCTION

Obesity is commonly considered a form of malnutrition that primarily emerged in the twientieth century, in association with lifestyles promoting high-energy intake or low physical activity [1, 2]. The influential ‘energy balance equation’ encourages us to consider the aetiology of obesity through the lens of ‘calorie counting’, and failure to regulate the balance between energy intake and expenditure [3]. The primary drivers are considered the food environment, marketing of unhealthy foods and beverages, urbanization and sedentary behaviour [2, 3].

Nevertheless, obesity was not uncommon among wealthier groups in the nineteenth century [4], and portraits and accounts of historical figures such as the British monarch Henry VIIIth document it in the sixteenth century [5]. Moreover, it was sufficiently common in ancient Greek, Roman, and Byzantine societies that physicians developed guidelines for clinical treatment [6, 7].

The further we look back into the human past, however, the more the literature highlights food scarcity and undernutrition as the primary nutritional stresses [1, 8, 9], even though the emergence of agriculture appears to have worsened the risk of famine [10] and was associated with an increase in skeletal markers of malnutrition in early European farmers [11]. Accordingly, it is rare for obesity to be presented as a pre-agricultural phenomenon. Since adiposity is a soft tissue trait, obesity cannot be directly preserved in the Palaeolithic fossil record. A candidate indirect marker is Diffuse Idiopathic Skeletal Hyperostosis (DISH), which links skeletal and metabolic abnormalities. Although DISH is more common among those with obesity, the great majority of individuals with obesity do not demonstrate DISH, especially at younger adult age [12]. Therefore, investigating DISH in the Palaeolithic skeletal record cannot provide reliable evidence of the occurrence or prevalence of obesity.

Instead, evolutionary perspectives on obesity have primarily focussed on how past selective pressures, including undernutrition, may have shaped the emergence of genetic variability that influences obesity susceptibility in contemporary settings.

For example, the influential ‘thrifty genotype’ hypothesis assumed that some ancestral populations were exposed to cycles of ‘feast and famine’ [8], selecting for the capacity to store fat rapidly through insulin resistance [13]. In ‘good times’, individuals with putative thrifty genes could accumulate fat reserves, subsequently drawing on them during ‘tough times’. Through such metabolic dynamics, individuals with thrifty genes could have been better able to accommodate short-term fluctuations in energy supply [9]. While this hypothesis has stimulated the field of obesity science, supporting evidence remains weak [14].

An alternative ‘drifty genotype’ hypothesis assumes that in recent human evolution, social strategies and tools increased the capacity to resist predation. In turn, this could have relaxed selective pressures on maintaining body weight within a range that enabled escape from predators [15], thus allowing new genetic variants associated with higher weight to accumulate in the gene pool. In modern obesogenic settings, these variants may explain individual variability in obesity susceptibility [16].

Neither the ‘thrifty genotype’ nor ‘drifty genotype’ hypothesis assumes that individuals in pre-agricultural environments were actually obese, or indeed that obesity carried fitness benefits in comparison with lower body weights [17]. Others have argued that selection favoured greater adiposity after the emergence of agriculture [18]. For example, the increased burden of infections in dense sedentary farming communities may favour storing fat when the energy supply permits [19].

Yet, there is one source of information that reliably indicates that Palaeolithic obesity *did* occur, moreover across a relatively wide geographical area. Numerous statuettes of the female form, many of them indicating obesity, have been recovered from western Eurasia [20, 21]. As they represent females, they are widely known as Venus figurines. These depictions, while stylized, are sufficiently accurate in terms of the anatomical distribution of body weight that it seems beyond question that multiple sculptors had observed actual female obesity. They are also sufficiently varied that they appear not to represent copies of a single figurine. Up until now, the primary interest in these figurines was in understanding what the artists meant to convey symbolically, a topic that remains controversial [20, 22, 23].

From a nutritional perspective, however, a striking question relates to the interactions of biological mechanisms with environmental drivers that could have led to high body weights in the Palaeolithic, moreover in a period of major nutritional stress. In the discussion that follows, we do not assume that Palaeolithic obesity was common, rather it is likely to have been rare, thus stimulating the sculpting of the figurines. We aim simply to explore potential pathways through which high body weight could have emerged. Drawing on mechanistic models that go beyond simple ‘calorie counting’, we consider how metabolism may be perturbed by interactions between developmental exposures and ecological stresses, which could have propagated effects across generations.

## VENUS FIGURINES

Venus figurines comprise a collection of small, sculpted representations of the female form, recovered from various European sites dating primarily from the Gravettian period of the Upper Palaeolithic. Most have been dated between 23 and 25 000 years BP, though a few are older (~38 000) or younger (14 000) [21, 24]. They are variously manufactured from stone, ivory, clay, or bone [21].

**Figure 1.**
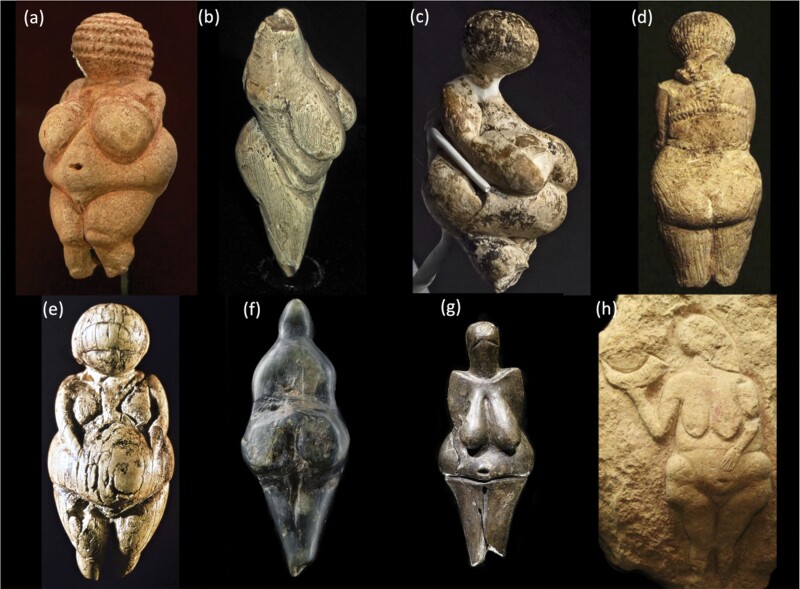
Examples of Venus figurines. (a) Venus of Willendorf, Natural History Museum in Vienna, carved from limestone. Austria (25–30 000 BP). Wikimedia Commons CC BY-SA 4.0 Credit: Jakub Hałun. (b) Moravany Venus, made of mammoth ivory tusk. Slovakia (22 000 BP). Credit: Don Hitchcock, donsmaps.com. (c) Venus from Gagarino, made of mammoth ivory tusk. Russia (21–20 000 BP). Wikimedia Commons CC BY-SA 4.0 Credit: Thilo Parg. (d) Venus made of limestone, Kostenki 1 site. Russia (21–23 000 BP). Credit: Don Hitchcock, donsmaps.com. (e) Venus made of mammoth tusk, Kostenki 1 site. Russia (22 000 BP). Wikimedia Commons CC BY-SA 4.0 Credit: C. Cohen. (f) Green steatite female figurine ‘Venus de Losange’ from Balzi Rossi. Italy (–19 000 BP). Credit: RMN-Grand Palais (Musée d’Archéologie Nationale), Jean-Gilles Berizzi. (g) Věstonická Venus at the Mammoth Hunters exhibition in the National Museum in Prague. Fired ceramic. Czech Republic (31–27 000 BP). Wikimedia Commons CC BY-SA 2.5 Credit: Petr Novák. (h) Venus of Laussel, carved into limestone rock. France (18-20 000 BP). Wikimedia Commons CC BY-SA 3.0 Credit: 120.

Although varying in many ways, the figurines also have some common features. In particular, the majority show extreme corpulence of the body, with little attention paid to the head or feet [25]. They are often naked, though some show indications of clothing. Most appear to be women of childbearing age, with some appearing relatively young, and several possibly indicating pregnancy. Examples of slim figurines are less common and come disproportionately from a few sites, at one of which they were recently shown to depict children and men as well as women [26]. Figure 1 illustrates a number of figurines from different European locations, all having in common not only large dimensions of anatomical regions associated with female fat storage but also fat-folds on the torso and limbs that are characteristic of human obesity.

What these figurines were intended to convey to the viewer remains uncertain. They may have represented fertility symbols, and potentially had religious significance [20]. Recently, researchers have considered their shape from a Darwinian perspective. The deliberate portrayal of large size during the Ice Age may indicate a specific link between harsh ecological conditions and adiposity [27]. Johnston *et al*. found that Venus figurines were manufactured during periods when the ice sheets were advancing, likely to have caused nutritional stress and increased risk of starvation. Moreover, the figures showed a trend of greater corpulence as the glaciers advanced, and a reverse trend as they retreated [27]. The authors concluded that the figurines conveyed a ‘physical message’ that high levels of body fat could promote survival, with this message being amplified during the most threatening periods. Given that body fat is a vital source of metabolic substrate during lactation [28], the statuettes might also have indicated the value of fatness for female reproductive fitness [27].

While these hypotheses are very plausible, little attention has been paid to how individual women might have achieved high body fatness in this challenging period.

## AN ECO-LIFE-COURSE MODEL OF OBESITY

Until recently, the primary aetiological framework used to explain the development of obesity was the energy balance equation, attributing excess weight gain to an excess of energy intake relative to expenditure.


$$\begin{array}{l} Change\;in\;weight=Energy\;intake\;energy\;expenditure. \end{array}$$

A dominant theme in obesity epidemiology has been to assess whether weight gain occurs because energy intake is ‘excessive’, or energy expenditure (e.g. on physical activity) ‘inadequate’ [29]. In public health, this has translated into an unfortunate and inappropriate tendency to attribute obesity to ‘gluttony’ or ‘sloth’ [3], thus ignoring how the drivers of obesity may be in the environment rather than the individual [30, 31]. If we projected this framework onto Palaeolithic hunter-gatherers, we might suggest that an extended seasonal glut of food, which might coincide with low levels of energy expenditure on foraging, could drive weight gain. In Western Europe, for example, steep-sided valleys could have acted as conduits of large herds of migrating animals carrying high body fat reserves for winter, providing a major seasonal food surplus for strategically placed human hunters [32].

However, there is increasing appreciation that while the energy balance equation is mathematically correct, being based on fundamental physics, it offers no genuine explanation for the cumulative weight gain that leads to obesity [33, 34]. Precisely because the equation is a truism, it fails to address the question, *why is there a persistent imbalance* between intake and expenditure, so that weight gain continues over long periods and obesity follows?

An alternative explanatory framework can be termed the ‘metabolic perturbation’ model, which assumes that diverse factors can impact metabolism at the cellular level and potentially drive fat deposition [33–36]. In this framework, it is the metabolic perturbation that causes lipogenesis, and positive energy balance is a symptom rather than the cause of weight gain. Accordingly, we should be looking not for markers of high-energy intake or low-energy expenditure, but rather for environmental factors that perturb cellular metabolism and appetite [34]. This issue is explored further below.

In applying this framework to the Venus figurines, we focus on three specific factors, which we selected as good candidates for helping explain paleo-obesity. Our interest in these factors relates to the fact that two of them can potentially be linked with paleo-data on the environment. Clearly, a wide range of other factors may have contributed to high body weight, and our aim here is simply to start the discussion and stimulate further exploration.

First, we explore the interaction of body composition with the thermal environment. Second, we focus on the associations of diet composition with weight gain. Third, we develop a life-course perspective that can help understand intergenerational effects. These approaches can be linked in order to reconsider the large body size indicated by the Venus figurines.

### Thermal environment and body composition

Variation in body shape and composition appears to be a key strategy whereby humans adapt to contrasting thermal environments [37, 38]. Among non-western populations measured from the mid-twentieth century onwards, with little technical ability to control the thermal environment, both lean mass and fat mass were found to scale inversely with mean annual temperature [39].

At a functional level, colder temperatures may favour greater lean mass for heat production. Morphological characteristics indicate that greater muscle mass helped Neanderthal populations adapt to cold environments [40]; however, this then had implications for energy requirements, for example, the Neanderthal diet may have needed to supply 3360 to 4480 kcal/day [40].

Cold environments may also favour greater adiposity as a source of energy to buffer temporary shortfalls in the food supply. In mammals generally [41] and humans specifically [42], basal metabolism is higher in colder environments, indicating that oxidizing a given mass of fat will meet energy needs for less time in the cold. However, one study reported relatively low subcutaneous fat in an Inuit population in the Arctic [43], suggesting that increased adiposity is not obligatory in colder settings and that low temperatures alone could not explain the large size of the Venus figurines.

Nonetheless, storing fat is widely recognized as a mammalian survival strategy [44], and low fatness predicts mortality in famines, as indicated by the worse survival of men compared to women [45, 46]. In colder environments, contemporary human populations show greater sexual dimorphism, with males disproportionately increasing in lean mass and females in fat mass [47]. This contrast may be due to the importance of female body fat as a supply of energy for lactation [28] and might help explain the disproportionate portrayal of women among the Venus figurines.

Overall, colder environments favour larger lean mass, while greater fat stores may also benefit fitness, especially in females. Increases in both lean and fat tissue will increase the girths of the body relative to height, but on their own, cold temperatures do not drive obesity.

### Diet composition and obesity

For decades, elevated adiposity was attributed to dietary intake of fat, the most energy-dense macronutrient [48, 49]. Protein was known to be the most satiating nutrient, fat intake was considered to drive excess weight gain, and high-carbohydrate low-fat diets were thus considered the solution [48]. Paradoxically, however, obesity prevalence in high-income countries increased over the decades when this framework dominated [36].

Recent work challenges this approach. First, increasing attention has been paid to refined dietary carbohydrate, following recognition of its link with insulin, the primary anabolic hormone [33–35]. Second, the framework of nutritional geometry broke new ground in proposing that the primary appetite of most mammals is not for energy, but for protein [50]. Satisfying the appetite for protein then influences dietary energy intake: calories are drawn in as protein needs are met (‘protein leverage’), and low-protein diets can lead causally to overconsumption of calories [50].

**Figure 2.**
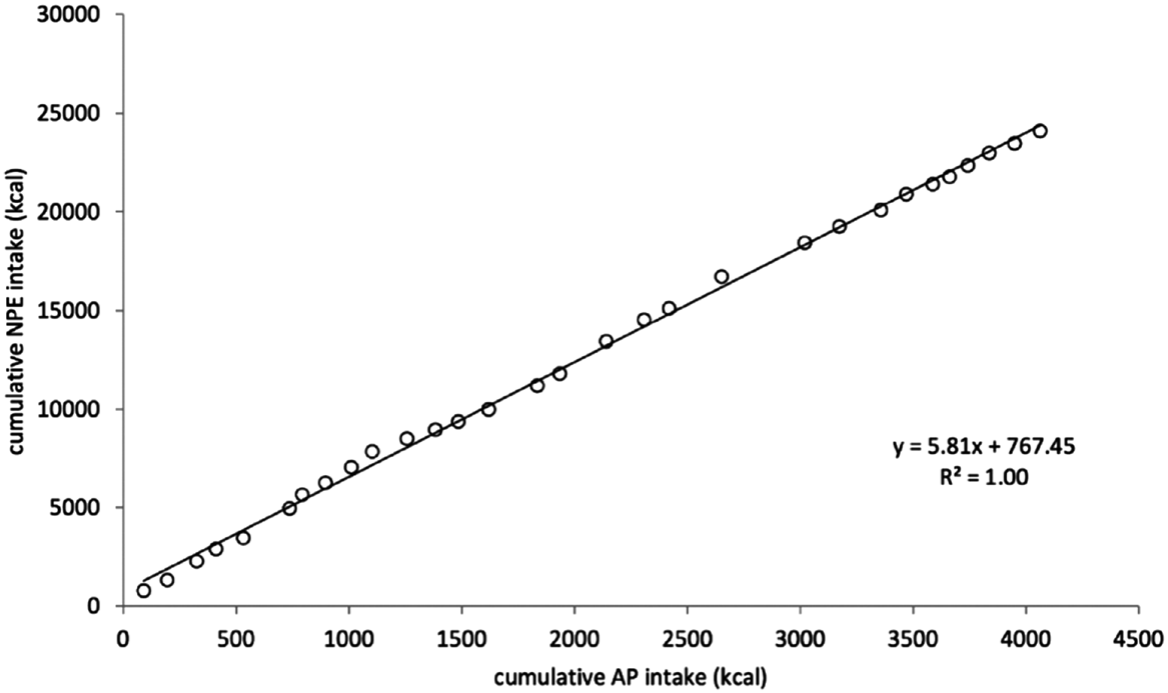
Balanced protein energy intake in a chacma baboon in the Cape Peninsula of South Africa. The figure plots the cumulative intake of non-protein energy in kcal against cumulative available protein in kcal over a 30-day period. Reproduced with permission from Johnson et al [52].

Observational and experimental studies support the ‘protein-leverage hypothesis’ in many species of insects and non-human mammals, though there are occasional exceptions [51]. Detailed study of a female baboon over 30 days showed striking constancy of the proportion of calories from protein in total calorie intake (**Fig. 2**), indicating a specific appetite for protein [52]. In humans, experimental reduction of dietary protein leads to subsequent compensatory increases, confirming a protein appetite [53]. The available evidence suggests that protein leverage of energy intake is incomplete in humans [54], but also strong enough to drive weight gain and contribute to the obesity epidemic [55, 56]. Importantly, a key determinant of protein requirement is lean body mass [57]. On this basis, individuals with higher lean mass might respond more strongly to a low-protein high-energy diet, leading to greater energy overconsumption.

### Developmental origins of obesity

The life-course perspective focuses on how metabolic responses to diet are shaped by exposures earlier in life, particularly those during foetal life that are shaped by maternal pregnancy physiology [58, 59].

A pioneering study linked foetal exposure to maternal famine with increased obesity risk in men aged 19 years, but only if the exposure occurred during the first trimester; for men exposed in the third trimester, adult obesity risk was actually reduced compared to those unexposed [60]. However, a follow-up study at 50 years of age failed to confirm these findings for men, rather it was women who had higher body mass index (BMI) and waist circumference if exposed to maternal famine in the first trimester [61]. Other retrospective studies of maternal famine exposure also report heterogeneous findings [62–64]. More reliable evidence comes from a prospective study, linking foetal growth faltering in the first trimester with higher birth weight compared to those not faltering [65]. However, these data are best considered to relate only to the developmental origins of obesity *susceptibility*. Whether or not obesity subsequently develops depends on the postnatal environment.

Contrasting with maternal famine, maternal obesity during pregnancy is reliably associated with offspring adiposity. Mechanistically, this may involve excess fuel transfer to the foetus [66, 67], changes in breastmilk composition [68], or epigenetic effects [69, 70].

Glucose is the key nutrient meeting foetal energy demand, and enabling foetal biosynthesis of molecules such as glycogen and fatty acids. If placental transfer exceeds demand, then glucose-derived energy is stored as foetal triglycerides synthesized from fatty acids. Both glucose transfer and triglyceride synthesis are under the control of foetal insulin [71, 72] that can already be elevated in the second trimester [73]. The result of foetal hyperglycaemia–hyperinsulinemia is elevated neonatal adiposity, and increased risk of obesity in childhood [74]. While hormones are key mediators, transmitted maternal alleles also explain some of the associations between maternal and offspring adiposity [75].

Importantly, maternal obesity and diabetes during pregnancy may continue to influence the offspring after birth. Foetal hyperinsulinemia induces methylation changes in the insulin receptor promotor in arcuate neurons of the hypothalamus, leading to receptor downregulation [76]. As a result, insulin-induced satiety signals originating from the hypothalamus are attenuated [77], increasing consumption of breast milk [78]. Breastmilk composition of macromolecules, metabolites, and hormones is also altered by maternal obesity, particularly in colostrum and transitional milk [79]. These mechanisms propagate longer-term effects so that exposure to maternal gestational diabetes is associated with persistent excess weight gain through childhood [80].

Overall, these mechanisms may, given appropriate environmental energy availability, allow the transmission of obesity susceptibility from mother to offspring. Although many studies compare mothers with obesity to those without, more detailed studies show dose–response associations of maternal metabolic phenotype with offspring adiposity [81]. Higher weight in the maternal generation may therefore influence how the offspring responds to its own nutritional environment. We now consider how these mechanisms might interact with protein leverage in a cold temperature setting.

## REVERSE-ENGINEERING PALEO-OBESITY

Linking the life-course and metabolic frameworks described above, we propose a composite hypothesis: that foetal exposure to maternal phenotype, interacting with chronic exposure to a low-protein diet in a cold-exposed population with high protein needs, may have promoted high body weight in a subset of susceptible individuals. The composite effects of these mechanisms could amplify across successive generations.

### Cold climate, high lean mass, and protein needs

As lean mass scales inversely with temperature in contemporary humans [39], we predict that Palaeolithic European populations inhabiting regions exposed to the Ice Ages demonstrated high lean mass. Greater lean mass would itself make larger fat depots more noticeable in terms of shape. Moreover, it would increase daily protein needs, which could have been harder to maintain during periods of dietary stress associated with the Ice Ages. On this basis, Palaeolithic foragers may have been inherently susceptible to the protein leverage of calories, as reviewed below.

### Dietary trends

Reconstructing ancestral diets is notoriously challenging. Contemporary hunting and gathering populations pursue a diversity of diets, regarding the relative contributions of animal versus plant foods and hence dietary macronutrient composition. This suggests that the *Homo* genus has no specific dietary niche, but can rather meet its nutritional requirements in different ways [82, 83]. Contemporary meat consumption appears especially variable, ranging from 5% to 90% of total energy intake, though populations inhabiting colder environments tend to have higher intakes [82]. However, the key tenet of our hypothesis is that dietary protein content *systematically declined* over the Palaeolithic, and specifically between the Aurignacian and Gravettian periods, when the Venus figurines were primarily produced.

At a broader level, any diet can be framed in terms of its trophic level, in other words, its position in the food chain that represents an ascending hierarchy of primary producers, herbivores, and primary or secondary carnivores [84]. Evidence from diverse sources indicates that the hominin lineage shifted from a low trophic base to a high carnivorous position during the Pleistocene [85]. Early hominin diets, including those of most *Australopithecus*, were predominantly plant-based [86–88], whereas from ~2.5 mya fossil evidence for late *Australopithecus* and early *Homo* indicates a shift towards meat consumption, and the origins of a hunting/scavenging and gathering subsistence pattern [82]. This trend towards carnivory may have peaked with *Homo erectus*, such that from the Middle and Upper Palaeolithic onwards, the trophic level may have declined again, reflecting a shift towards more omnivorous and varied diets [85].

Stable isotope data from enamel and bone collagen can indicate the dietary source of protein, differentiating whether the main source was marine or terrestrial (13C) and of plant or animal origin (15N). The available evidence indicates that most European Upper Palaeolithic populations were consistently consuming terrestrial animal protein [89, 90]. However, this method provides no information on the proportion of protein in total energy intake, and thus cannot evaluate the dilution of protein by non-protein energy, as specified in the protein leverage hypothesis.

High meat consumption among Middle and Upper Palaeolithic populations is indicated by several sources of evidence, including anthropogenic behaviour preserved on faunal remains, stone tool residues, sophisticated stone tool production and stable isotope data, which demonstrate both a dietary overlap with wolves and other carnivores, but also dissimilarities with their prey [91–97]. Nevertheless, grinding tools for plant processing, including the production of flour, have been recovered at 30 000-year-old sites in Europe [70]. Moreover, direct evidence of plant food consumption is preserved in plant micro-remains lodged in dental calculus and microwear damage on the enamel surface of teeth from the hard exteriors of plant foods, combined with the abrasive action of inadvertent grit introduced into the oral cavity. These remains include starches and phytoliths, which are taxon-specific [98, 99]. Dental calculus is thus a principal source of knowledge about dietary proclivities [100].

Prior to the appearance of Upper Palaeolithic modern humans in Europe, the dietary staples of Neandertals were grass seeds and underground storage organs alongside other plants [101]. Even during extreme Ice Ages such as the end of Marine Isotope Stage 4 some 60 000 years before present (BP), where reindeer was the animal most often hunted, Neandertals still consumed starches from grass seeds and underground storage organs as evidenced by the plant micro-remains preserved in the dental calculus of an older adult female, La Quina 5 (98). Although this individual had a weak plant food signal, she still managed to consume complex carbohydrates, even in this formidable Ice Age habitat [102].

Dental microwear texture analysis captures the surface complexity of the areas of the molars that occlude during mastication when the teeth grind against one another to crush and pulverize the food bolus [103–108]. One variable shown to differentiate diets comprising hard and brittle versus softer foods is Area Scale Fractal Complexity (asfc), while another, known as anisotropy (or exact proportion length-scale anisotropy of relief, epLsar), separates farmers with monotonous diets from foragers with diverse diets [107–109]. Plants contain hard parts, such as seeds, seed coats, shells, phytoliths, and mechanically resistant exterior tissues along with adhering grit, which effectively puncture and scratch the occlusal surface, separating the protein bonds of the enamel matrix. Grit derived from grinding plant foods with stone tools may elevate asfc values.

**Figure 3.**
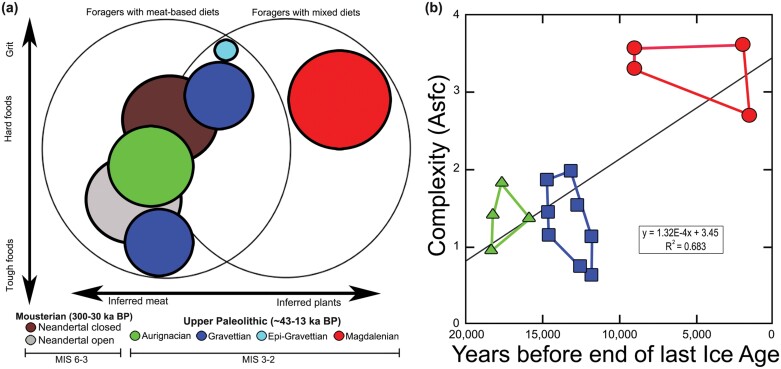
Dietary trends towards plant-based diets, indicating lower protein content. (a) A Venn Diagram shows overlap between recent human foragers with meat-based diets and those with plant-based diets compared to Middle Palaeolithic Neandertals and Upper Palaeolithic modern humans, demonstrating an increase in the use of plants, particularly in the late Upper Palaeolithic (Magdalenian); Marine Isotope Stage (MIS) 6–3 ranges from 195 to 30 000 BP, while MIS 3–2 is equivalent to 60–13 000 BP. Source data from El Zaatari and Hublin [110]. Figure adapted with permission from Power and Williams [102]. (b) Complexity (asfc) compared to years before the end of the last Ice Age (Pleistocene) approximately 13 000 BP, demarcated by the onset of the Younger Dryas, for the Aurignacian (triangles), Gravettian (squares) and Magdalenian (circles), each with a convex hull inclusive of 100% of the variation for the technocomplex. A linear regression of these two variables yields a significant constant (*P* < .001) and coefficient (*P* = .002) and a relatively tight fit (*R*^2^ = 0.683, *n* = 17) between inferred plant use (complexity) and years before the end of the last Ice Age. This analysis included Magdalenian sites, such as Farincourt 1 (14 000–15 000 years BP) [111]; Saint-Germain La Rivière (between 15 780 (± 200) and 14 100 (±160) years BP) [112], Abri Pataud 1 (22 000 years BP) [113], Lachaud 3 (described as proto-Magdalenian II, based on comparisons of regional stone tool types; estimated at 22 000 years BP) [114]; Gravettian sites including Dolní Věstonice 13 and 15 (27 640 years BP) [115], Dolní Věstonice 16 (25 740 years BP) [116], Dolní Věstonice 31 (between 29 300 and 25 820 years BP) [117], Pavlov 1 (26 170 years BP) [117], Předmostí 1 (between 24 340 (±120) BP and 26 780 (±140) years BP) [118], Cro-Magnon 2 (27 680 years BP) [119], Barma Grande 1 and 2 (24 800 years BP) [120], and Aurignacian sites, including Les Rois R50 #31 (27 270 to 30 440 years BP) [121] and Mladeč 1, 2, and 8 (31 190, 31 320, and 30 680 years BP, respectively) [122]; complexity data from El Zaatari and Hublin [110].

These two variables can be quantified for numerous Neandertal and Upper Palaeolithic foragers, primarily 125–13 000 years BP. Higher complexity values from later compared to earlier Upper Palaeolithic sites (Fig. 3a) imply an uptick in plant food consumption around the coldest interval of the last glacial maximum [100]. This dietary shift is reflected in the increased use of food processing to render plant foods digestible by cooking, pounding, chopping, and later by mixing, brining, and fermenting [100].

### Chronology

The earliest Upper Palaeolithic stone tool tradition of Europe, which signals the arrival of modern humans, is the Aurignacian, initially appearing at 43–40 000 BP, followed by the Gravettian, evident at ~35 000 BP. The Gravettian coincides with the beginning of the last glacial maximum, which built to a peak over 10–15 000 years, and is associated with some of the most exquisite Venus figurines. Around the peak at ~21 000 BP, a technologically sophisticated culture appears, the Magdalenian, occurring approximately 20–13 000 BP.

Dietary signals differed between earlier and later Upper Palaeolithic sites [110]. Analysis of asfc values for Aurignacian, Gravettian, and Magdalenian sites shows a significant increase in plant use as the last Ice Age proceeded [106] (Fig. 3b). Looking in more detail, the transition from the Aurignacian to the Gravettian was associated with stable asfc values, whereas epLsar values increased by 45% [110]. Conversely, the transition from Gravettian to Magdalenian was associated with 130% increase in asfc values but a decline in epLsar values. Also of relevance is the contrast between sites characterized by forest-steppe versus open-steppe, the latter more representative of advancing ice sheets. Open-steppe sites had 53% higher asfc values, but 8% lower epLsar values.

Both of these analyses are limited by very small sample sizes, and most differences are not statistically significant. Nevertheless, although the two markers show different patterns, the observations suggest that plant foods, such as grass seeds and underground storage organs, were already appearing in Gravettian diets, and that plant foods may also have increased in colder settings over this period. On the assumption that plant foods have lower protein content than meat, these dietary trends indicate a cumulative dilution of dietary protein availability during the period when the Venus figurines were produced.

### Skeletal evidence

Another indirect marker of changing protein intake comprises changes in height and lean mass. In contemporary humans, global epidemiological studies indicate that low animal protein intake inhibits linear growth [123], while smaller-scale studies show reduced height in vegan and vegetarian children compared to omnivores [124, 125]

Consistent with these patterns, studies have indicated downward trends in skeletal components of size in the Palaeolithic [126, 127], which might indicate reduced protein and/or energy availability. Data from the early Upper Palaeolithic indicate extremely tall heights, though extrapolating from individual bone lengths to total stature is challenging. Whatever equations are used to generate total stature, however, the bioarchaeological record indicates a substantial reduction in height through the Palaeolithic of around 10 cm in both sexes, with associated declines in body weight [127–129]. Intriguingly, the loss of height co-occurred with secular increases in limb robusticity, indicating that later populations had more demanding subsistence activities, itself a marker of greater use of plant resources [128]. Glaciations were not the only stress over this period, hence other ecological drivers might have contributed, and some of the decline in stature may have reflected genetic change in association with population dynamics [130].

A more specific change was a reduction in leg length relative to total stature [127], and a number of studies have illustrated developmental links between short stature and obesity [131–133], mediated by reductions in the capacity for fat oxidation that appear to manifest in adult women but not men [132, 134]. Moreover, as predicted by thermodynamic theory [135], shorter legs reduce heat loss to the environment and hence promote adaptation to colder conditions [136].

These trends may therefore reflect simultaneously adaptation to colder temperatures, but also developmental changes favouring fat storage over height growth, especially in women. Both of these adaptive trends could have been driven in part by reduced protein content in the diet. Although the overall trend appears to be a decline in weight, the evidence from the Venus figurines indicates that in some individuals, dietary energy availability was sufficient to enable high body weight.

**Figure 4.**
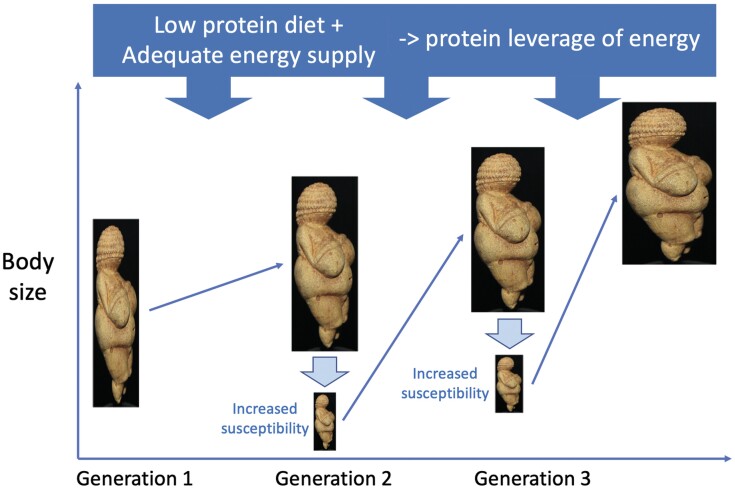
Schematic diagram illustrating the composite eco-life-course conceptual model for the emergence of paleo-obesity across successive generations in a period of nutritional stress (declining protein availability). A low-protein diet, accompanied by adequate overall energy supply, favours weight gain within the life-course. Through the impact of high body weight and adiposity on pregnancy physiology, the susceptibility to gain weight on exposure to a low-protein diet may be increased in the offspring, resulting in morbid obesity emerging cumulatively across generations. Original image of Venus of Willendorf, Wikimedia Commons CC BY-SA 4.0 Credit: Jakub Hałun. Adaptations of image by author JW to simulate inter-generational trends.

These effects could then have amplified across generations, as higher levels of maternal weight generated a metabolic niche during pregnancy that made the subsequent generation more sensitive to protein leverage, thus stimulating intergenerational trends towards obesity in the most susceptible individuals (Fig. 4). Individual genetic variability, as highlighted in the drifty genotype hypothesis [15], may also have contributed.

### Maternal obesity as a selective pressure

Contemporary physiological mechanisms may also indicate the presence of obesity in ancestral populations. Through the lens of ‘parent-offspring conflict theory’, the placenta has been proposed to act in foetal interests under the stress of nutritional scarcity, in order to extract more nutrients from maternal metabolism [137]. However, several sources of evidence indicate that the placenta may also play a role in protecting the foetus from excessive maternal nutrient transfer, which can occur when the mother has obesity and/or diabetes during pregnancy [138]. This protective capacity indicates that maternal overnutrition acted as a selective pressure with some frequency in the past, though exactly when is unknown.

## LIMITATIONS

**Figure 5.**
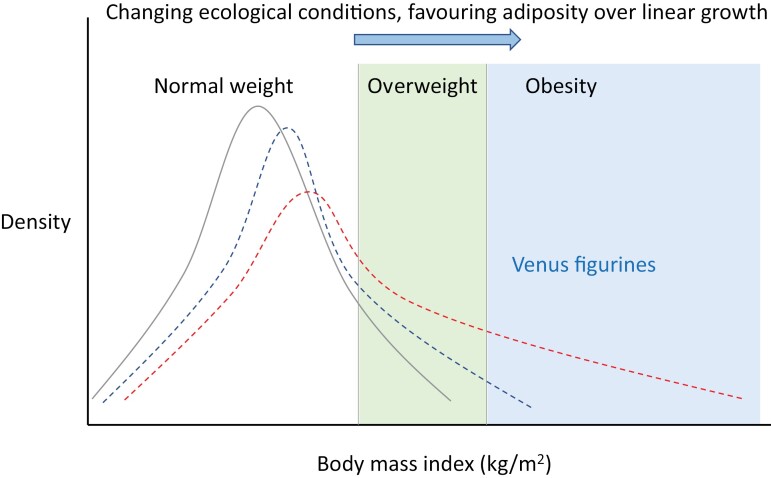
Alternative population distributions of BMI. Changes in ecological conditions increase not only the median BMI but also the degree of right-skew, as some individuals respond more strongly than others to the new conditions. This pushes a small number of individuals over the threshold for obesity, and larger numbers in to the category of overweight.

There are undoubtedly challenges in attempting to obtain biological insight from works of art, that are idealized images. We consider that the anatomical accuracy of the fat-folds on many figurines indicates that artists in different times and geographical places had seen human obesity. However, the fact that individual archaeological sites often had several Venus figurines does not tell us anything about the prevalence of obesity. In contemporary populations, the prevalence of obesity reflects changes in the population distribution of BMI. Figure 5 illustrates how changes in ecological conditions can generate small increases in median BMI, accompanied by increasing right skew, driving an increasing proportion of the population above the thresholds of overweight and obesity. Artists may well have focussed on those with the highest weights, but it is likely that larger numbers may have achieved overweight, while others remained thin. The notion that height may decline even as adiposity increases is consistent with life history theory [139], that is, the reallocation of metabolic resources away from linear growth to energy reserves, and has been observed in many studies of life-course adaptation across species [132, 140–142], as well as inferred at the origins of agriculture [143].

Since there are very few statuettes of males, the hypothesis that we have developed relates only to females. Currently, obesity tends to be more common in women than in men in low-income countries, with this sex difference attenuating in middle-income countries and disappearing in high-income countries [144, 145]. We suggest that any evidence for female obesity in the Palaeolithic does not reliably indicate whether male obesity also occurred, though as discussed below, risk factors may also differ by sex. The dental evidence of dietary protein change from Aurignacian to Gravettian is also inconsistent, depending on the marker considered. It is hoped that richer datasets may in the future provide more robust evidence for the trends and mechanisms that we hypothesize.

An alternative framework for understanding dietary constraints and obesity is the insurance hypothesis, which assumes that organisms increase their body fat stores when food supply becomes unpredictable [146, 147]. In humans, this hypothesis has been used to explore associations of household food insecurity with overweight and obesity. In high-income countries, this hypothesis is supported for adult women but not men, whereas in low-income countries, the association does not hold, most likely because food insecurity may overlap with energy scarcity [147]. However, the insurance and protein leverage hypotheses need not be mutually exclusive. In the USA and Brazil, food insecurity has been associated with a greater intake of ultra-processed foods [148, 149], which, in turn, reduces dietary protein content [150]. In rural India, food insecurity was similarly associated with both obesity and low dietary protein in women [151]. More generally, many populations worldwide are experiencing a dual burden of malnutrition, where poor quality diets are associated with both stunting (short stature) and overweight [152].

## CONCLUSION

We have suggested an eco-life-course conceptual model that could potentially account for some women developing obesity prior to the agricultural era, moreover during a period of nutritional and environmental stress. We consider the Venus figurines, sculpted by Palaeolithic artists with their own interests in large body size, to be the only available evidence of a more general trade-off between height and adiposity in the Gravettian period. Our hypothesis links exposure to a low-protein diet in a cold setting with intergenerational dynamics that could amplify the consequences of metabolic adjustments through effects on pregnancy physiology. We do not argue that obesity was common in any population, rather the mechanisms we describe may have interacted with genetic susceptibility as in contemporary populations [15, 153], altering the population distribution of nutritional status. Our approach breaks new ground by presenting changes in protein availability, rather than energy supply, as the relevant nutritional stress.

**Figure 6.**
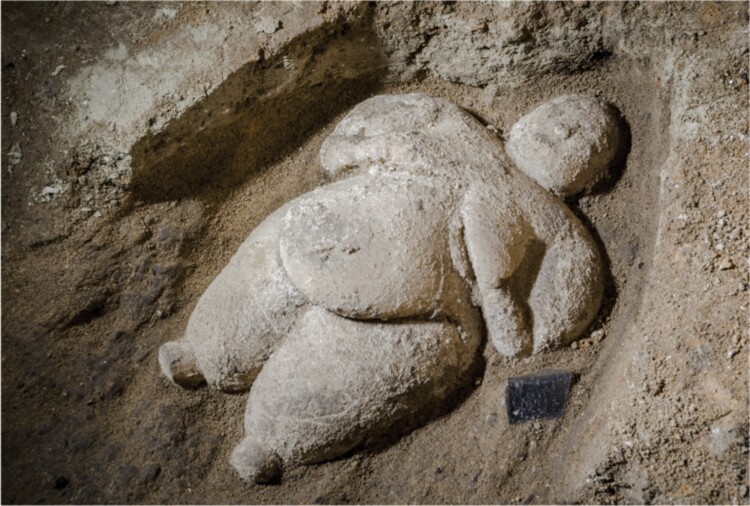
A female figurine of large size from the early Neolithic site of Çatalhöyük in southern Anatolia, dating from 5500 to 8000 BC. Image by Jason Quinlan reproduced with permission from the Çatalhöyük Research Project.

Importantly, Venus figurines by no means vanished as populations began to adopt agriculture. Large quantities of female figurines expressing large body size have been recovered from the Neolithic site of Çatalhöyük from the southern Anatolian plateau in Turkey (Fig. 6), from the period 7400 to 6200 BCE [154]. In this setting, new cereal crops such as wheat, barley, and rye came to dominate the diet, though animal proteins from many sources were also consumed, and hunting remained an important food source [155]. Our hypothesis suggests that this population was likewise adapted to a high-protein diet and that the introduction of cereal crops may have simultaneously diluted protein availability while also providing greater non-protein energy which could, through protein leverage, have favoured excess weight gain in some individuals. Venus figurines persist in other places in the Near East and central Europe throughout the early Neolithic [156].

In setting out this conceptual framework, we acknowledge that the supporting evidence is currently limited and has inconsistencies. We hope that our approach will stimulate bioarchaeologists and paleoanthropologists to investigate paleo-obesity in more detail and to draw on our understanding of human biology to interpret the evidence.

## Data Availability

No data were produced specifically for this review. The source data for Figure 3 are specified in the figure legend and bibliography.
